# The Long-Term Consumption of Ginseng Extract Reduces the Susceptibility of Intermediate-Aged Hearts to Acute Ischemia Reperfusion Injury

**DOI:** 10.1371/journal.pone.0144733

**Published:** 2015-12-09

**Authors:** Pei Luo, Gengting Dong, Liang Liu, Hua Zhou

**Affiliations:** 1 State Key Laboratory of Quality Research in Chinese Medicine, Macau University of Science and Technology, Avenida Wai Long, Taipa, Macau, China; 2 School of Chinese Medicine, Hong Kong Baptist University, Kowloon Tong, Kowloon, Hong Kong, China; Indiana University School of Medicine, UNITED STATES

## Abstract

**Background:**

A large number of experimental studies using young adult subjects have shown that ginseng (*Panax ginseng* C.A. Meyer) protects against ischemia heart disease. However, ginseng has not been explored for its anti-I/R effect and mechanism of action in the aged myocardium. The present study was designed to evaluate the effects of the long-term consumption of ginseng extract on myocardial I/R in an *in vivo* rat model and explore the potential underlying mechanism.

**Methods and Results:**

Young (6-mo-old) and intermediate-aged (18-mo-old) rats were gavaged with either standardized ginseng extract (RSE) at 80 mg/kg or vehicle for 90 days. The rats were sacrificed after LAD coronary artery ligation was performed to induce 30 min of ischemia, followed by 90 min of reperfusion. The myocardial infarct size was measured. Left ventricular function was evaluated using pressure-volume loops. The levels of survival, apoptotic and longevity protein expression were assessed through Western blot analysis. Myocardial pathology was detected through H&E or Masson’s trichrome staining. We observed higher infarct expansion with impairment in the LV functional parameters, such as LVSP and LVEDP, in aged rats compared with young rats. Enhanced Akt phosphorylation and eNOS expression in RSE-treated aged hearts were accompanied with reduced infarct size, improved cardiac performance, and inducted survival signals. In contrast, p-Erk and caspase 7 were significantly downregulated in aged rats, suggesting that cardiomyocyte apoptosis was suppressed after RSE treatment. RSE also inhibited caspase-3/7 activation and decreased Bax/Bcl-2 ratio. Consistent with the results of apoptosis, Sirt1 and Sirt3 were significantly increased in the RSE-treated aged heart compared with vehicle-treated I/R, suggesting that the anti-aging effect was correlated with the anti-apoptotic activity of RSE.

**Conclusion:**

These findings suggest that the long-term consumption of ginseng extract reduced the susceptibility of intermediate-aged hearts to acute ischemia reperfusion injury in rats. These effects might be mediated through the activation of Akt/eNOS, suppression of Erk/caspase 7 and upregulation of Sirt1 and Sirt3 in intermediate-aged rats.

## Introduction

Ginseng, the dried root of *Panax ginseng* C.A. Meyer, is cultivated in China, Korea, Japan, and Russia. In Asian countries, ginseng has been used as a treatment for various illnesses and as a daily supplement for over 2000 years [1]. A large number of experimental studies show the protective effect of ginseng against myocardial ischemia/reperfusion (I/R) injury, and clinical reports also support the cardiovascular benefits of this treatment [2, 3]. Despite knowledge of the broad cardiovascular effects of ginseng, several issues remained unresolved. Among these, the different therapeutic outcomes between young and aged subjects remain elusive. According to the records of traditional Chinese medicine, ginseng is more suitable for aged than for young individuals. While the cardioprotection-related effects of ginseng were established from studies using young animals or normal cells, there are no reports regarding the use of this medicinal root in aging hearts. The results of a previous study on the cardioprotective effects of ginseng strongly suggested that a chemically standardized ginseng extract RSE has remarkable anti-I/R injury effects [4]. However, there is little evidence as to whether and how RSE pretreatment conferred protection against I/R injury in *in vivo* models of the aged myocardium.

Acute myocardial infarction (MI) is the leading cause of heart failure and cardiac mortality, particularly in the aged people. The risk and prevalence of acute MI progressively increases with age [5]. Aging manifests as detrimental alterations in heart pathological outcomes, including cardiomyocyte cell loss, myocardial hypertrophy, and collagen deposition. These normal aging changes do not necessarily contribute to morbidity, but they are clearly associated with the decline in cardiac function observed with aging, such as the lengthening of contraction and relaxation, decreased heart rate and reduced cardiac output. Therefore, the higher mortality resulting from MI in aged people, partially reflects altered heart function. Cardiac aging in rodent models from childhood to the aged adults recapitulates the aging-related alternations in human hearts [6, 7]. Aging rat hearts revealed the age-dependent impairment of systolic and diastolic function, compatible with the finding that aging hearts are more susceptible to ischemic injury. However, despite prospective clinical studies on individuals in three age groups, young (under age 19), intermediate (age 19–64) and aged (age 65 and over), the drug therapeutic mechanism responsible for the age-related risk of cardiac infarction remains unclear. Molecular mechanisms underlying I/R injury are complex, including ion channels, reactive oxygen species, inflammation, endothelial dysfunction, mitochondrial abnormalities, cardiomyocyte apoptosis and necrosis[8]. Studies by us suggest that the cardioprotective actions of ginseng are probably mediated by hormone receptors and PI3K/Akt/eNOS pathway [4].

We proposed that the use of intermediate-aged SD rats might alter responses to ischemic reperfusion injury, and long-term RSE treatment might play a similar role as observed in the middle-age population, in which clinical myocardium infarction occurred. The increased myocardial infarction in middle-aged SD rats (18 month old) at the time of infarction compared with that in young 6-month-old rats has not been confirmed using experimental *in vivo* models, and the mechanism responsible for the increased susceptibility of intermediate-aged hearts to MI remains completely unknown. We compared the age-related difference in cardiac function and pathological changes responsible for the increased susceptibility to myocardial ischemia in intermediate-aged hearts following acute I/R injury. I/R injury typically develops in the rat left anterior descending (LAD) ligation model, representing an opportunity for examining this important issue. The present study was designed to examine the effects of the long-term consumption of ginseng extract on the functional and morphological changes of the myocardium in naturally middle-aged rats. Herein, we investigated 1) differences between young and intermediate-aged rats after RSE treatment for 90 days in the occurrence of infarct size and left ventricular (LV) function following LAD ligation-induced MI and 2) potential mechanisms responsible for the age-related differences observed after ginseng treatment.

## Materials and Methods

### Animals

Male Sprague-Dawley rats were purchased from the Laboratory Animal Services Center, the Chinese University of Hong Kong, Hong Kong. The animals were acclimated for 7 days under a 12-hour light/12-hour dark cycle at room temperature (22°C ± 1°C). A chow diet and water were provided ad libitum. Animal care and treatment procedures were performed in accordance with the Institutional Guidelines and Animal Ordinance (Department of Health, Hong Kong Special Administrative Region) and approved through the Committee on the Use of Human and Animal Subjects in Teaching and Research of the Hong Kong Baptist University.

### Materials

The standardized ginseng extract (RSE) was prepared using the dried root of *P*. *ginseng* C.A. Meyer through ethanol extraction. The contents of the ginsenosides Rg1, Re, Rb1, Rc, Rb2, Rd, Rk3, (20S)Rg3, (20R)Rg3, Rk1 and Rg5 were 8.08, 7.64, 11.58, 10.35, 6.67, 4.50, 0.06, 0.29, 0.09, 0.08, and 0.10 mg/g, respectively, determined through HPLC analysis. Dorminal (20%; 1 ml contains 200 mg pentobarbital sodium) was purchased from Alfasan, and 2, 3, 5-triphenyltetrazolium chloride (1612634) was obtained from International Laboratory USA (San Bruno, CA, USA). Other materials used in this study are specified in detail in the sections below.

### Experiment design

**Fig 1:**
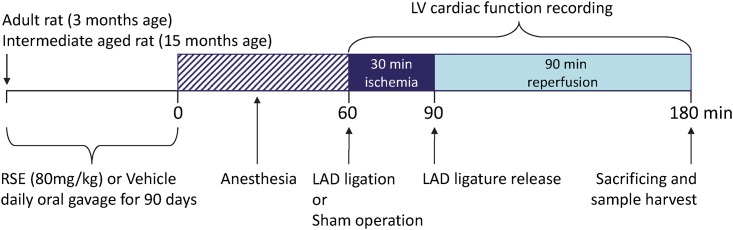
Outline of the experimental protocol. The experimental protocol used to evaluate the anti-aging effects of RSE against myocardial ischemia and reperfusion (I/R) injury. Young and intermediate-aged rats were fed RSE (80 mg/kg) or vehicle once daily for 90 days. After the last administration on the 90th day, the rats were subjected to LAD occlusion for 30 min ischemia, followed by 90 min reperfusion.

Thirty young rats at the age of 3 months and thirty intermediate-aged rats at the age of 15 months were randomized into six groups, respectively, 10 rats for each group as follows: 1) young vehicle-treatment group with sham operation; 2) young vehicle-treatment group with I/R; 3) young RSE-treatment group with I/R; 4) intermediate-aged vehicle-treatment group with sham operation; 5) intermediate-aged vehicle-treatment group with I/R; 6) intermediate-aged RSE-treatment group with I/R. The rats are treated either with RSE (dissolved in distilled water at 10mg/ml) at a dosage of 80 mg/kg/day or with vehicle (distilled water at 8ml/kg) through gavage for 90 days as indicated in the group information. On Day 90, the rats underwent sham operation or myocardial I/R (I/R). Of 10 rats in each group, 6 were for ventricular function and infarct size measurement and 4 for western blot analysis and histological assessments. Myocardial I/R was induced through LAD coronary artery ligation. Briefly, under anesthesia with sodium pentobarbital (70 mg/kg body weight) and artificial ventilation (rodent ventilator IITC SAR 830/P), the heart was exposed via left lateral thoracotomy, followed by pericardiectomy. The LAD coronary artery was occluded using a 6–0 silk suture and a small vinyl tube. Ischemia was established by tightening the suture from both ends with fixed weight. The rats subsequently underwent 30 min of ischemia and 90 min of reperfusion through the gentle release of the snare. The sham operation was conducted using the entire surgical protocol described above, without the introduction of LAD ligation and release. An outline of the experimental protocol is shown in Fig 1.

### Analysis of myocardial infarction

Myocardial infarction was analyzed according to heart infarct size determination as previously described (Zhou, et al., 2011). The rat hearts were reperfused for 90 min, the left ventricle was cut perpendicular to the base-apex axis into six 2–3 mm slices, followed by staining with 1% TTC solution. The images of the slice were captured using a LEICA digital camera 480, and the infarct area in each slice was measured using computed planimetry with the image analysis program ImageJ 1.26 (Wayne Rasband, National Institutes of Health, Bethesda, MD, USA). The myocardial infarct size was calculated by dividing the total infarct weight of each left ventricle by the total weight of the ventricle.

### Cardiac function assessment

A Millar P-V catheter (SPR-838, Millar Instruments, Inc., Houston, TX, USA) was inserted into the left ventricular cavity via the right carotid artery. The femoral artery was isolated and cannulated using a PE 50 polyethylene tube connected to a physiological pressure transducer (SP 844, MEMSCAP, Crolles Cedex, France). By PowerLab (ADInstruments Pty Ltd., Castle Hill, Australia), the left ventricle pressure and mean aortic pressure (MAP) were recorded using a Millar catheter and physiological pressure transducer, respectively. Electrocardiogram (ECG) in lead II was also recorded through the needle electrodes attached to the limbs. The left ventricular end diastolic pressure (LVEDP), left ventricular systolic pressure (LVSP), maximum values within a beat of the first derivative of left ventricular pressure (+dp/dt), and heart rate (HR) was calculated from a continuously generated pressure signal for 15min stabilization. The LV P-V loop was acquired using a Millar control unit (MPVS-300/400 system, Millar Instruments, Houston, TX, USA) by transiently compressing the inferior vena cava for 3s-period. The volume calibration of the conductance system was performed as previously described [9]. Briefly, the volume calibration of the catheter was performed using small tubes of known diameters (a calibration cuvette supplied by Millar Instruments) filled with fresh, slightly heparinized blood. In this calibration, the linear volume-conductance regression of the absolute volume in each cylinder versus the raw signal acquired from the conductance catheter was used as the volume calibration formula. All analyses were performed using the Millar analysis software PVAN 3.4 (Millar Instruments, Houston, TX, USA) according to the manufacturer’s instructions.

### Caspase-3/7 activity measurement

One hundred milligrams of frozen left ventricular tissue was homogenized with cold buffer (0.1% TritonX-100, 2mM DTT, 5mM MgCl_2_, 25mM HEPES, pH7.5) and centrifuged at 12,000 × g for 30min at °C. The supernatants were collected and caspase-3/7 activity was determined using Apo-ONE Homogeneous Caspase-3/7 Assay kit (G7790, Promega) according to the manufacturer’s protocol.

### mRNA isolation, cDNA synthesis and quantitative real-time PCR

Total RNA was extracted from frozen left ventricular sample by Tissue Total RNA Mini kit (Favorprep, Taiwan) according to the manufacturer`s instruction.1μg RNA was reverse transcribed into cDNA by the transcriptor first strand cDNA synthesis kit (Roche, Basel, Switzerland). The amounts of cDNA were quantified using the SYBR Green I reagent (Roche, Basel, Switzerland) and detected by the Applied Biosystems ViiA^™^ 7 Real-Time PCR System (Applied Biosystems, Life technologies, NY, USA).

RT-PCR primers, Bcl-2: 5-GCGAAGTGCTATTGGTACCTG-3 (foward) and 5-ATATTTGTTTGGGGCAGGTCT-3 (reverse), Bax: 5-AGAGGCAGCGGCAGTGAT-3 (foward) and 5- AGACACAGTCCAAGGCAGCAG-3 (reverse), β-actin: 5-CTCTGTGTGGATTGGTGGCT-3(foward) and 5-GGGTGTAAAACGCAGCTCAG-3 (revers) from (TECH DRAGON LIMITED, Hong Kong, China). 2-ΔΔCT method was used to quantify gene expression levels.

### Western blot analysis

After I/R, the left ventricular samples were homogenized in a buffer containing 20 mM Tris/HCl, pH 6.8, 1 mM EDTA, 1% SDS, 1 mM PMSF, and 1X protease inhibitor cocktail (Roche, Germany). The cell lysates were immediately centrifuged at 12,000g for 10 minutes at 4°C and the supernatant was the cytosolic fraction. The mitochondrial and nuclear fractions were isolated according to the kit protocol of Qproteome^®^ Mitochondria Isolation kit (Qiagen, Hilden, Germany). The protein concentration of cytosolic fraction was quantified by Bio-Rad protein assay kit (Bradford method) (Bio-Rad, CA, USA). The protein concentration mitochondrial and nuclear fractions were determined by BCA protein assay kit (Thermo, Rockford, IL, USA). Equal amounts of proteins (40 μg/cytosolic fraction and 10 μg/ mitochondrial and nuclear fractions) were separated on a 10% SDS/PAGE and transferred onto nitrocellulose membranes (PALL BioTrace, New York, USA). The following antibodies were used to analyze the expression levels: Akt, phospho-Akt (Ser473), eNOS, Erk1/2 and phosphor-Erk1/2 (Thr202/Tyr204), Caspase 7 p20, Sirt1, Sirt3, Caspase 3, Bax, Bcl-2, β-actin, GAPDH, Histone and Cox 4 (Cell Signaling Technology, Danvers, MA, USA or Santa Cruz Biotechnology, Santa Cruz, CA, USA). The protein bands were detected using horseradish peroxidase conjugated secondary antibody (anti-mouse or anti-rabbit, Santa Cruz Biotechnology) and ECL reagent (Amersham Biosciences, Piscataway, NJ, USA) according to the manufacturer’s instructions. The densities of immunoreactive bands were quantified using image J software and normalized to loading control, respectively.

### Heart histological examination

The heart tissue was fixed in 10% formaldehyde at 4°C for at least 3 h and dissected into 5-μm-thick paraffin sections. The middle ventricular paraffin sections were stained with hematoxylin and eosin and Masson’s trichrome for collagenous fibrosis. Evidence of myocardial injury was evaluated by an investigator who was blinded to animal grouping according to a scoring system as described previously [10], i.e.: 0) nil; 1) minimum (focal myocytes damage); 2) mild (small multifocal degeneration with slight degree of inflammatory process); 3) moderate (extensive myofibrillar degeneration and/or diffuse inflammatory process); and 4) severe (necrosis with diffuse inflammatory process). The score from light microscopy observation was taken for statistical analysis with a nonparametric test.

### Measurement of Malondialdehyde (MDA) content

Frozen left ventricular tissue was homogenized and content of MDA was measured with a MDA assay kit (Nanjing Jiancheng Bioengineering Institute, Nanjing, China) according to the manufacturer’s instruction.

### Statistical analysis

The data are presented as the means±SD. The sample size of each group was 6 hearts for infarct size and cardiac function evaluation. The sample size of each group was 4 hearts for western blot, RT-PCR, histological score and caspase activity analysis. Differences of quantitative data among the groups were analyzed using one-way ANOVA. When the ANOVA showed an overall difference, post hoc contrasts were performed between groups using the SNK method. Comparisons of histological scores were analyzed by the non parametric Kruskal-Wallis test and pairwise differences by the Mann-Whitney test. The results were considered significant at p<0.05.

## Results

### Effects of RSE on myocardial infarct size

**Fig 2:**
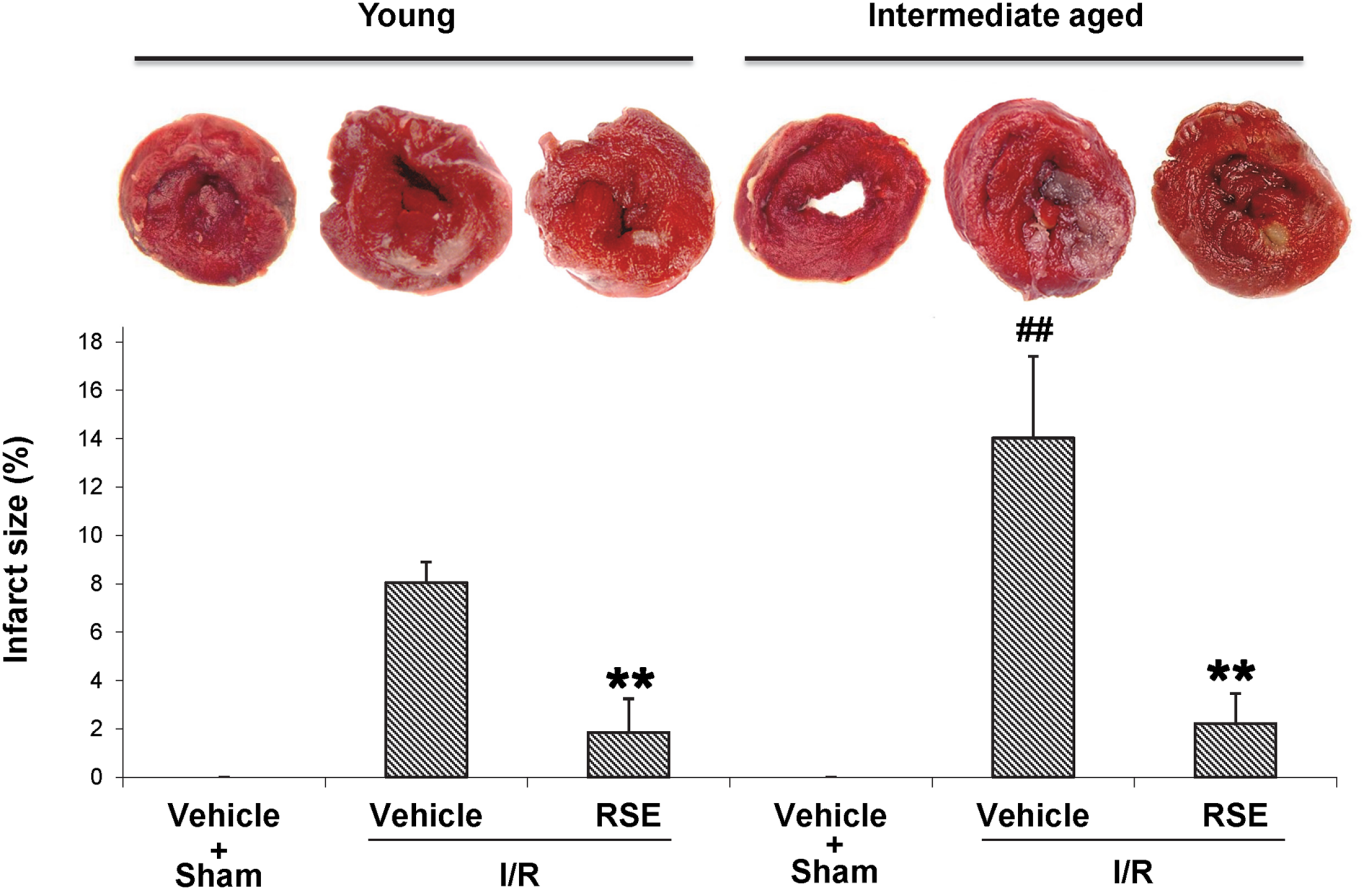
Myocardial infarct size determined using the TTC method. Young and intermediate-aged rats were fed RSE or vehicle as described in the protocol of Fig 1. After 30 min ischemia and 90 min reperfusion, the hearts were stained with TTC. The infarct size (%) was calculated as the ratio of the infarcted area (pale) to the risk area (deep-red). *Top*: representative photos of TTC stained left ventricle slices. *Bottom*: bar chart of myocardial infarct size determined through TTC staining. The data are shown as the means±SD, n = 6 rats/group. **P<0.01 represents vehicle-treatment with I/R in young and intermediate-aged rats, respectively. ##*P*<0.01 represents vehicle-treatment with I/R in young rats compared with vehicle-treatment with I/R in intermediate-aged rats.

In the same open chest model as described above, young rats and intermediate-aged rats received 30 min ischemia and 90 min reperfusion (I/R) or sham operation. As shown in Fig 2, the infarct sizes of the hearts in young and intermediate-aged rats treated with vehicle after I/R were 8.05±0.85% and 14.03±3.38%, respectively, suggesting that intermediate-aged rats showed significantly more severe I/R injury than young rats (P<0.01). RSE treatment for 90 days significantly reduced the myocardial infarct size compared with the respective vehicle control (1.85 ±1.39% in young rats and 2.22±1.24% in intermediate-aged rat, respectively). There was no significant difference in the infarct size between hearts treated with RSE in young or intermediate-aged rats.

### Effects of RSE on left ventricular function

**Fig 3:**
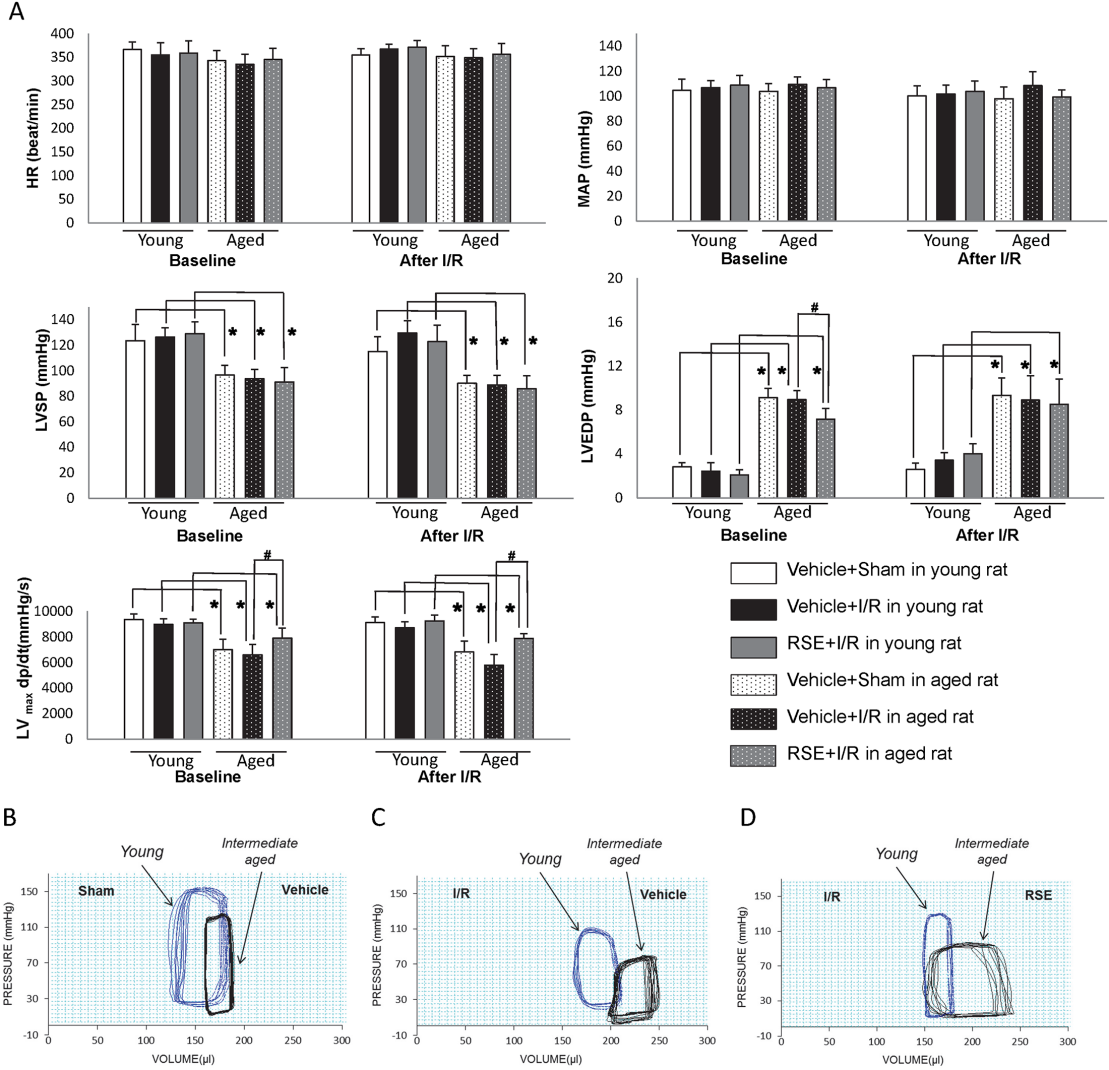
Effect of RSE on cardiac function. After 90 days, the rats were subjected to 30 min ischemia, followed by 90 min reperfusion. Left ventricular function was monitored at baseline and after reperfusion. (A) The data are shown as the means±SD, n = 6 rats/group. HR, heart rate; MAP, Mean arterial pressure; LVSP, left ventricular systolic pressure; LVEDP, left ventricular end-diastolic pressure; LV _max_ *dp/dt*, maximum first derivative of LVDP. *, *p* <0.05 vs. young, ^#^, *p* <0.05 vs. vehicle-treated aged. Representative pressure-volume (P-V) loops obtained using the Millar P-V conductance catheter system from vehicle-treated young and intermediate-aged rats underwent sham operation (B), vehicle-treated young and intermediate-aged rats subjected to I/R(C), RSE-treated young and intermediate-aged rats subjected to I/R (D).

There were no significant differences at baseline and after reperfusion in HR and MAP between the groups (Fig 3A). The RSE-treated hearts in both young and intermediate-aged rats did not significantly alter HR and MAP. Left ventricular function parameters LVSP, LVEDP and LV_max_ *dp/dt* for intermediate-aged hearts were appreciably changed. RSE treatment for 90 days in intermediate-aged hearts significantly reduced LVEDP and increased LV_max_ *dp/dt* (P<0.05), but didn’t change LVSP. In young hearts, there was no significant difference in LVSP, LVEDP and LVmax dp/dt either at baseline or after reperfusion. Fig 3B and 3C displays typical P-V loops obtained at baseline and after I/R in young and intermediate-aged rats. The shift of the P-V loops to the right in intermediate-aged rats indicated decreased cardiac contractility compared with young rats. The impaired ventricular relaxation and slightly increased end-diastolic stiffness were ameliorated after 90 days of RSE treatment. A characteristic continuous left shift in the P-V loops and an increase in the amplitude of the pressure signal indicated a smaller LV operating volume due to dilation of the chamber and an increase in contractility after I/R.

### Effects of RSE on p-Akt, Akt and eNOS expression

**Fig 4:**
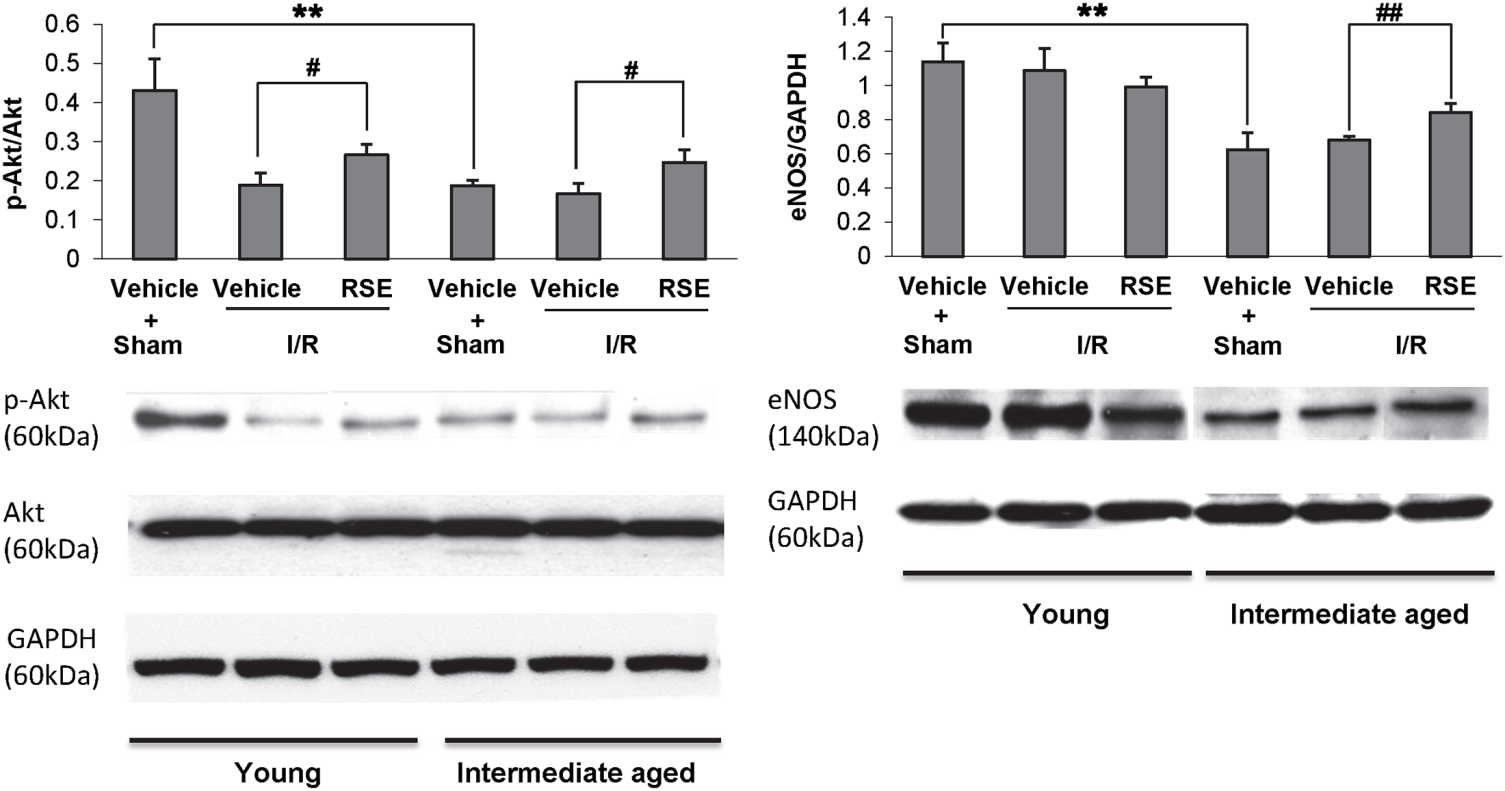
Western blot analysis of phospho-Akt, Akt and eNOS proteins in young and intermediate-aged rat hearts. GAPDH was used as the respective loading control. The bar charts represent quantitative comparisons between the groups. The relative density was shown as the means±SD, n = 4/group. ^#^ *P*<0.05, ** *P*<0.01.

Akt proteins in the heart were investigated after 30 min ischemia and 90 min reperfusion through LAD occlusion. As shown in Fig 4, total Akt was approximately equivalent in young and intermediate-aged rats treated with or without RSE. The phosphorylation of Akt(p-Akt) was significantly decreased in sham operated intermediate-aged rats, when compared to sham young rats. Thus, the p-Akt/Akt ratio was significantly reduced as a result of aging. I/R significantly suppressed the p-Akt, both in young and intermediate-aged rat hearts. Akt phosphorylation was significantly higher in hearts treated with RSE for 90 days compared with those treated with vehicle for 90 days. The expression of eNOS was significantly decreased in intermediate-aged hearts without I/R stimulus compared with those in young hearts with the sham operation. RSE treatment in intermediate-aged rats significantly enhanced the expression of eNOS compared with rats treated with vehicle (P<0.01).

### Effects of RSE on p-Erk, Erk and caspase 7 p20 expression

**Fig 5:**
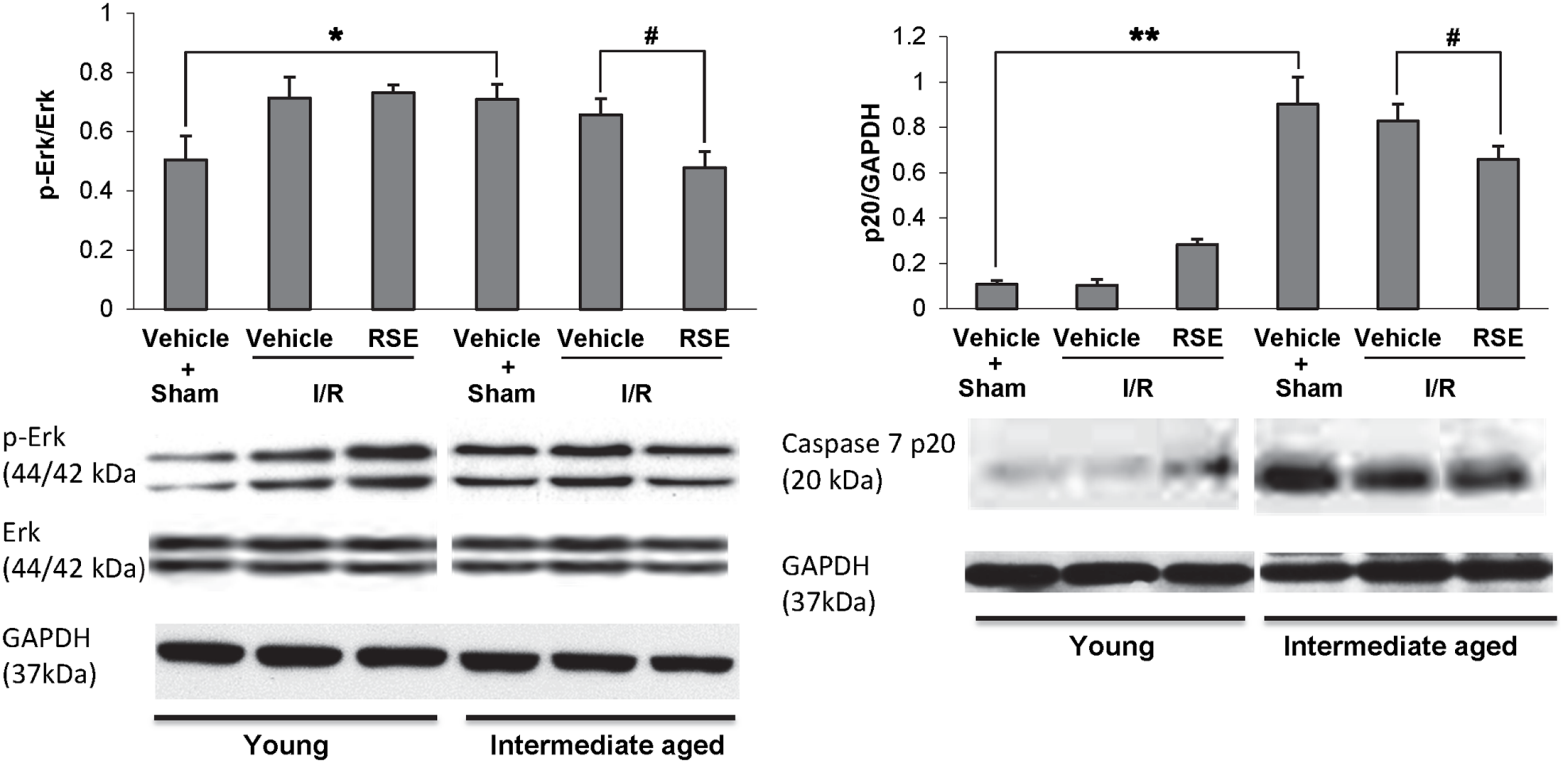
Western blot analysis of phospho-Erk, Erk and Caspase 7 p20 proteins in young and intermediate-aged rat hearts. GAPDH was used as a respective loading control. The bar charts represent the quantitative comparison between the groups. The relative density was shown as the means± SD, n = 4/group. ^#^ *P*<0.05, * *P*<0.05, ** *P*<0.01.

Because RSE induces the survival signals through the Akt/eNOS signaling pathway, we also investigated the activation of Erk with or without RSE treatment. Fig 5 shows the effects of RSE on the expression and activation of Erk. Total Erk was approximately equivalent in young and intermediate-aged rats treated with or without RSE. In hearts subjected to the sham operation without RSE treatment, the phosphorylation of Erk (p-Erk) was significantly enhanced in intermediate-aged rats compared with young rats (P<0.05). There was no difference in the p-Erk/ Erk ratio between vehicle and RSE-treated I/R hearts in young rats. Unlike young rats, the activation of Erk in intermediate-aged hearts with or without RSE treatment for 90 days showed a significant difference after 30 min ischemia and 90 min reperfusion through LAD occlusion. RSE reduced the p-Erk/ Erk ratio in intermediate-aged rats compared with those treated with vehicle (P<0.05). The expression of activated caspase-7 (p20) was markedly increased in intermediate-aged hearts compared with young rats (P<0.01). The expression of caspase-7 (p20) in young hearts without RSE after the sham operation or I/R did not significantly change. RSE treatment inhibited the expression of caspase-7 (p20) in intermediate-aged rats after I/R (P<0.05), implying that RSE likely protected the aging myocardium through the inhibition of Erk-caspase-7 activation.

### Effects of RSE on longevity proteins

**Fig 6:**
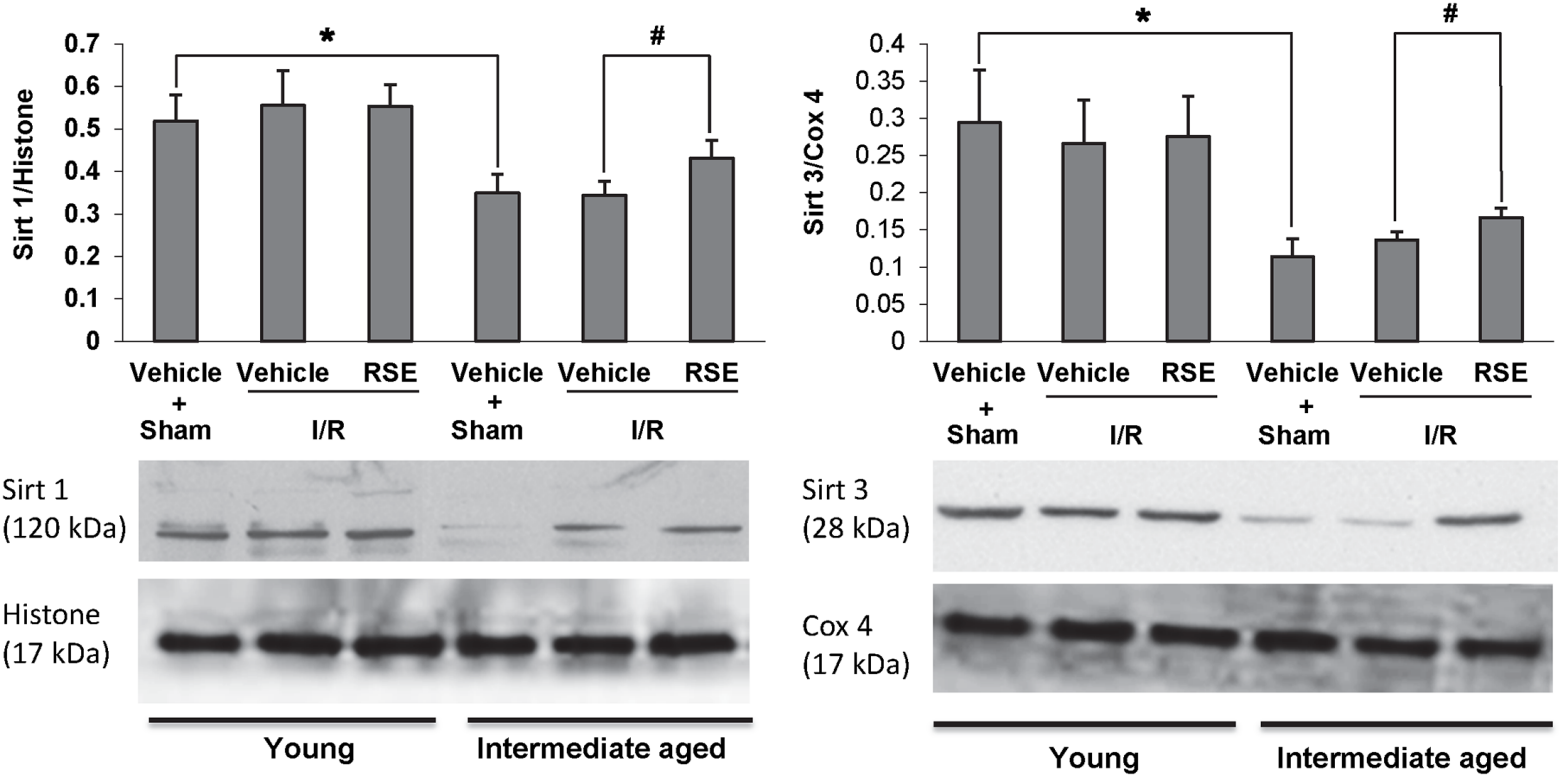
Western blot analysis of Sirt1 and Sirt3 protein expression in young and intermediate-aged rat hearts. Histone and Cox 4 were used as loading controls. The bar charts represent quantitative comparisons between the groups. Relative density was shown as the means± SD, n = 4/group. ^#^ *P*<0.05, * *P*<0.05.

The expression of the longevity proteins Sirt1 (nuclear) and Sirt3 (mitochondrial) was investigated in young and intermediate-aged rats. Fig 6 showed a significant difference in Sirt1 and Sirt3 expression in intermediate-aged rats compared with young rats (P<0.05). After I/R, Sirt1 and Sirt3 expression was significantly enhanced in the RSE-treated intermediate-aged heart compared with the respective vehicle-treated control (P<0.05).

### Effects of RSE on cardiac histological changes

**Fig 7:**
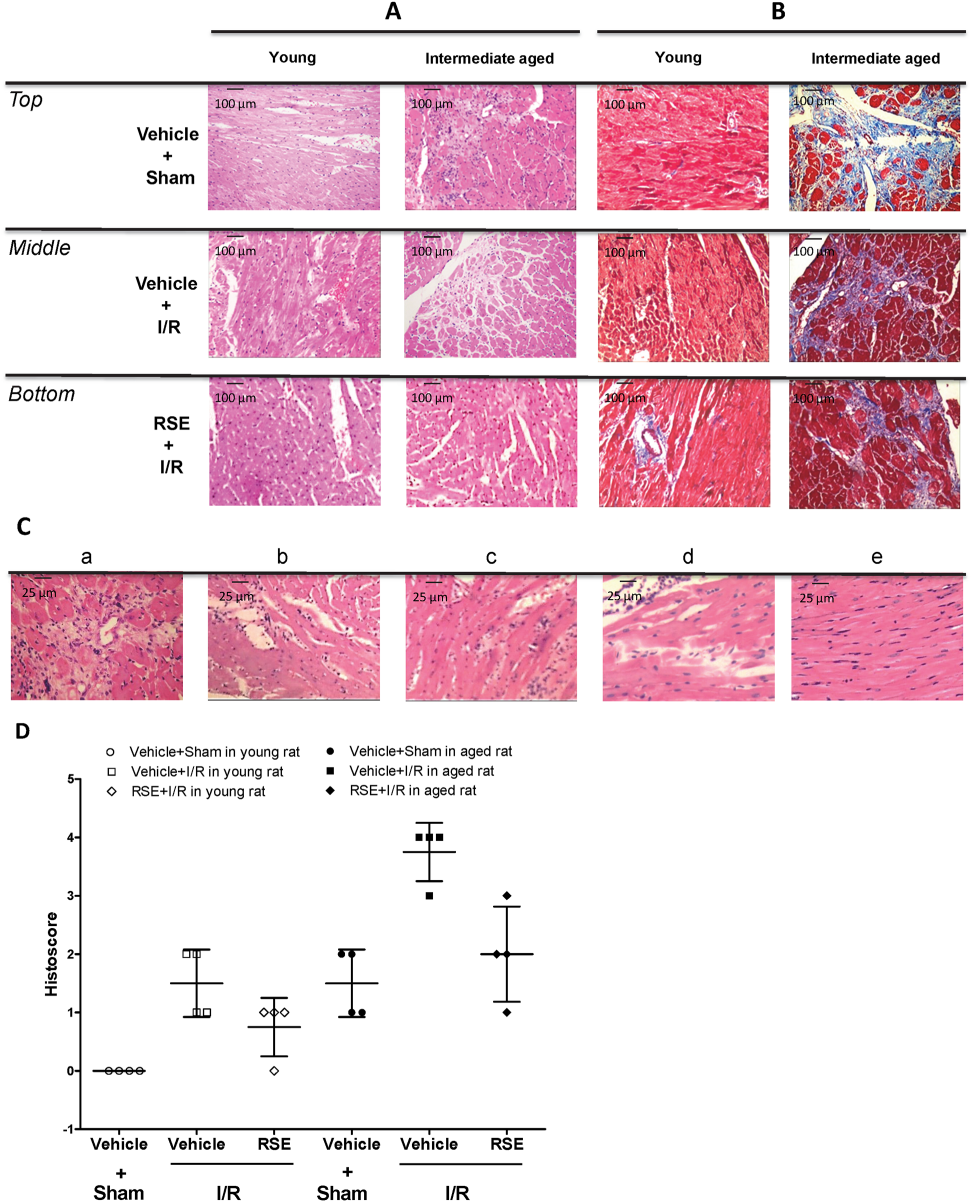
Histological characterization and collagenous fibrosis in the hearts of young and intermediate-aged rats. magnification × 4 (A and B) or × 10 (C)is shown. A. Representative images showing the effects of RSE on histological changes in the heart after I/R or sham operation (hematoxylin and eosin stain). B. Representative images showing the effects of RSE through collagenous fibrotic staining in the heart after I/R or sham operation (Masson’s trichrome staining). Photomicrograph showing the cross section of cardiac tissues in rats treated with vehicle after the sham operation (*Top*), vehicle-treatment after I/R (*Middle*) and RSE-treatment after the I/R operation (*Bottom*). C. Representative heart section with myocardial injury histoscore of 4 (a, severe), a histoscore of 3 (b, moderate), a histoscore of 2 (c, mild), a histoscore of 1 (d, minimum), and 0 (e, nil). D. Scatter plot of histoscore. n = 4 rats in each group.

Fig 7A shows the infarct and peri-infarct regions of young and intermediate-aged rats. In intermediate-aged rat hearts, these regions showed increased hyaline cytoplasm, vacuolized cytoplasm, variable hypertrophic myocyte fiber sizes, collapsed sarcomeres, and mineralization. A significant increase in the region of viable ventricular muscle was observed in the hearts of both young and intermediate-aged rats in the RSE-I/R groups compared with the vehicle-I/R groups. In accord with these findings, the histological scores analysis revealed a markedly enhanced I/R-induced myocardial injury in comparison with the control group. Fig 7B shows collagen staining and fibrosis in young and intermediate-aged hearts. No significant fibrosis in RSE- or vehicle-treated young hearts was observed. The fibrotic infiltration of the left ventricle was more concentrated in the endocardium with age. In intermediate-aged hearts, fibrosis was not significantly reduced in the RSE-treated I/R group compared with the respective vehicle-treated group. Fig 7C and 7D indicated that the highest level of histoscore was observed in the intermediate-aged I/R group, showing significant necrosis with diffuse inflammation. Treatment with RSE attenuated the severity of myocardial injury in the intermediate-aged hearts.

### Effects of RSE on activated Caspase-3/7 and Bax/Bcl-2 ratio

**Fig 8:**
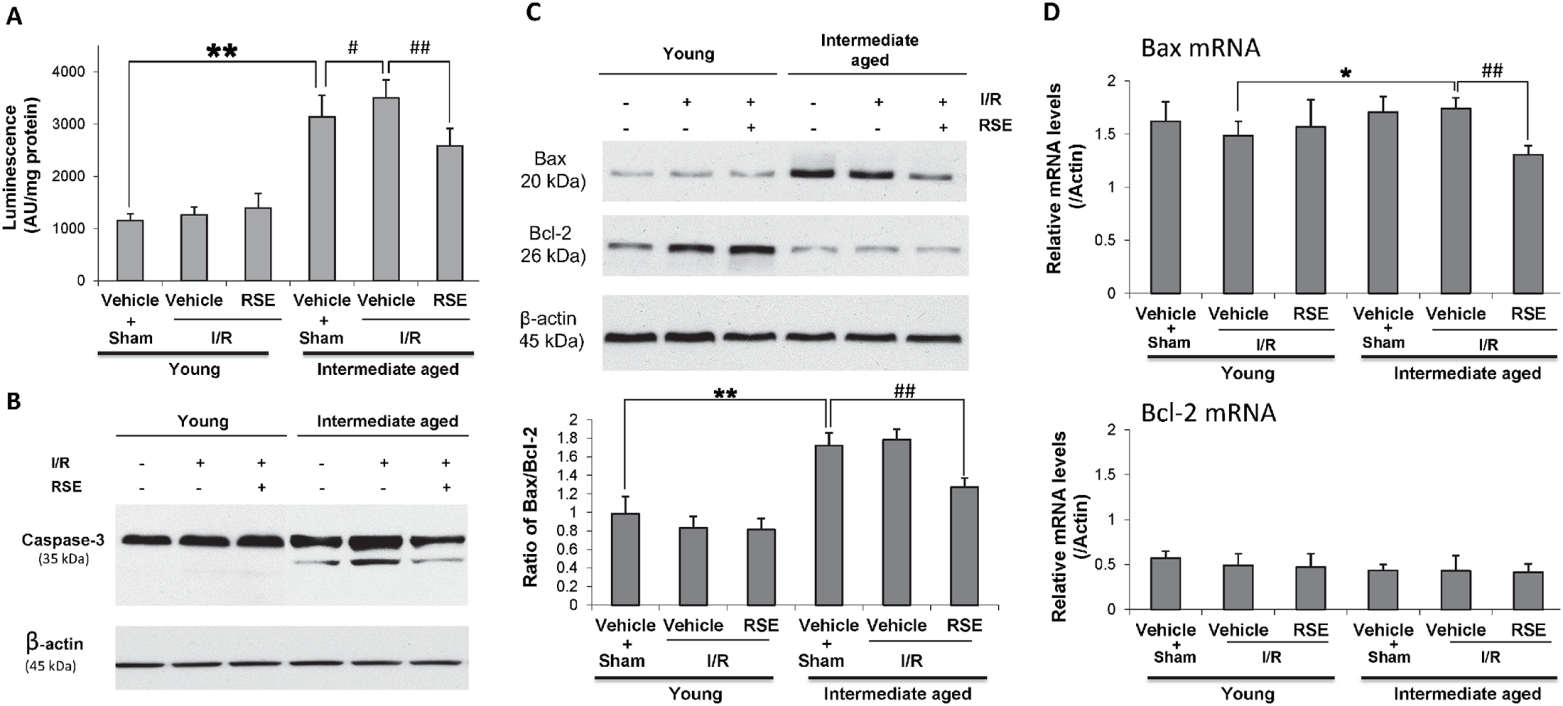
Evaluation of myocardial cell apoptosis induced by I/R in young and intermediate-aged rat hearts. A. Left ventricular tissue was lysed and then the caspase3/7 activity was measured as Luminescence signal. B. Caspase-3 processing was evaluated by Western blotting. C. Bax and Bcl-2 protein expression in young and intermediate-aged rat hearts. β-actin were used as loading controls. The bar charts represent quantitative comparisons between the groups. Bax/Bcl-2 ratio calculated from the density analysis. D. Bax and Bcl-2 mRNA expression was evaluated by quantitative real-time PCR. The values were shown as the means± SD, n = 4/group. ^#^ *P*<0.05, * *P*<0.05, ^##^ *P*<0.01, * *P<0.01.

Since one possible apoptotic pathway was the trigger of caspase cascade, we evaluated the Caspase-3/7 activity in the myocardial tissue. As shown in Fig 8A, caspase-3/7 activity showed no change in the young groups with or without RSE treatment, suggesting that the cell apoptosis elicited by I/R in young hearts was independent of the caspase cascade. Fig 8A showed a significant increase in caspase-3/7 activity in intermediate-aged rats compared with young rats (P<0.05). Interestingly, there was a significant increase in intermediate-aged rats after I/R when compared with Sham intermediate-aged rats. RSE treatment clearly suppressed the activated caspase-3/7 (P<0.05) that was accompanied with the reduction in the process of caspase-3 shown in Fig 8B. To further investigate the effect of RSE on apoptosis-regulatory proteins, we examined the protein and mRNA of anti-apoptosis marker Bcl-2 and pro-apoptosis marker Bax in the myocardial tissue. As shown in Fig 8C and 8D, Bax protein was increased in intermediate-aged hearts, whereas the protein expression of Bcl-2 was down-regulated, indicating a clear increased ratio of Bax to Bcl-2. RSE significantly suppressed Bax protein expression and decreased Bax/Bcl-2 ratio in intermediate-aged hearts compared with the vehicle treatment group. RSE treatment also significantly suppressed the mRNA of Bax induced by I/R in intermediate-aged rats. The mRNA levels of Bcl-2 showed no change in comparison with vehicle-treated intermediate-aged rats.

### Effects of RSE on myocardial MDA content

**Fig 9:**
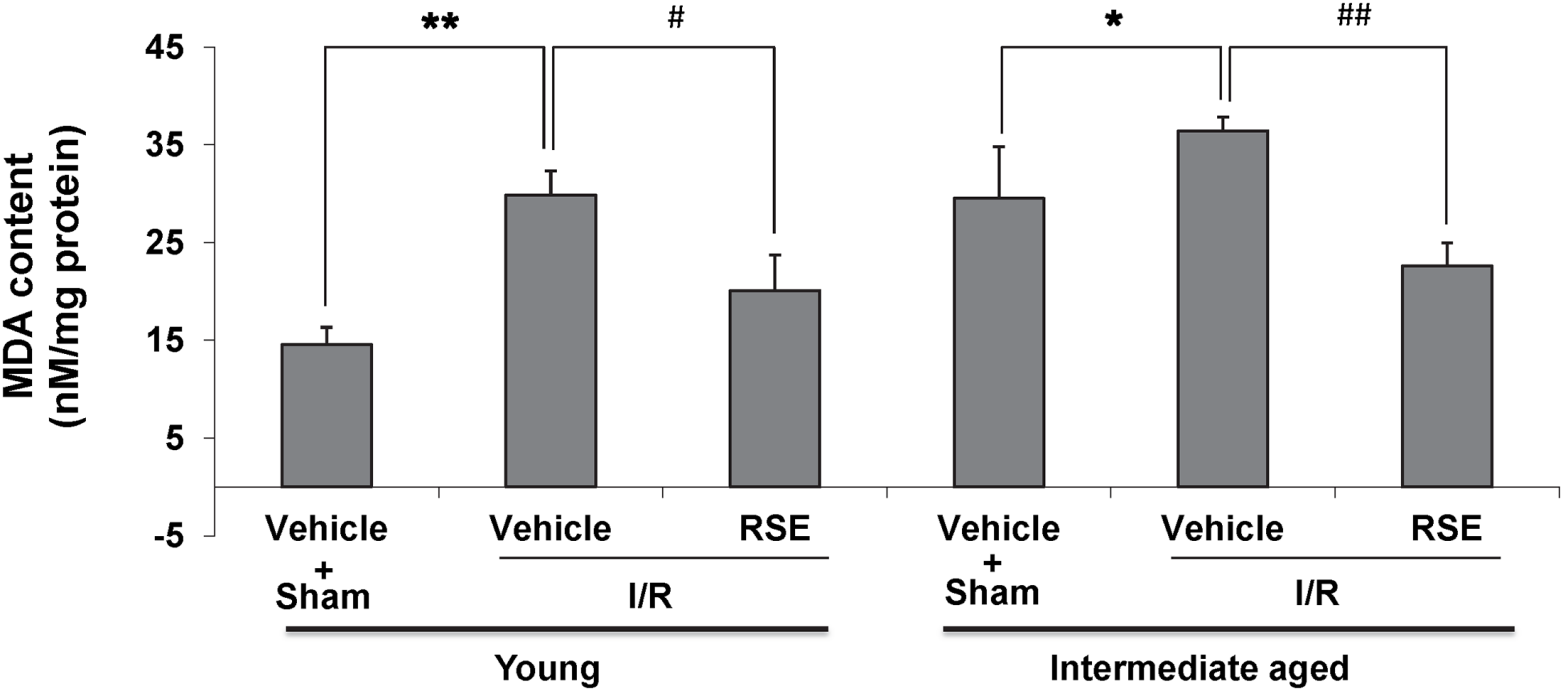
Measurement of MDA content in young and intermediate-aged rat hearts. Myocardial MDA levels were assayed using a commercially available kit. The values were shown as the means± SD, n = 4/group. ^#^ *P*<0.05, * *P*<0.05, ^##^ *P*<0.01, * *P<0.01.

MDA is biomarkers of ischemia stress in cardiomyocyte, so we measured the content of MDA in left ventricular tissue to evaluate changes in I/R injury. Fig 9. showed that I/R significantly increased content of MDA compared with the sham hearts (in young P<0.01, in intermediate-aged hearts P<0.05). RSE treatment significantly reduced the content of MDA compared with the vehicle treatment young hearts (P<0.05) or the vehicle treatment intermediate-aged hearts (P<0.01).

## Discussion

Intrinsic cardiac aging is defined as slowly progressive structural changes and functional declines with age in the absence of major cardiovascular risks. Aging makes the heart more susceptible to stress and contributes to increased cardiovascular mortality and morbidity in the elderly. The epidemiological analysis of patient age subsets showed that the in-hospital mortality of patients with acute myocardial infarction was only 2.5% for young patients compared with 9.0% in middle-aged patients and 21.4% in elderly patients [11]. A number of studies have revealed that the acute myocardial infarction in three age subsets was significantly different in risk factors, clinical features, morbidity and mortality [12, 13]. Although several studies have characterized the cardiovascular system in senescent rats (older than 24 months), only a few studies have examined cardiac function and structural alternations after I/R injury in middle-aged rats. In the present study, we directly compared the hearts from young and intermediate-aged SD rats and observed an aged-related increase in susceptibility to injury induced through ischemic reperfusion in middle-aged individuals. Using a rat model of LAD ligation, we studied the importance of middle age in the risk of myocardial infarction following I/R and explored the potential mechanism. We showed that 1) intermediate-aged rats had significantly higher infarct expansion than young rats after I/R; 2) left ventricular dysfunction were more severe in intermediate-aged rats compared with young rats; 3) a significant decrease in the levels of phosphorylated Akt and eNOS and the increased expression of phosphorylated Erk and caspase 7 were observed in intermediate-aged rats compared with young rats; 4) lower levels of the longevity proteins Sirt1 and Sirt3 were shown in intermediate-aged rats, indicating a stronger aging response; 5) activation of caspase-3/7 and increase of Bax/Bcl-2 ratio were observed in intermediate-aged rats, revealing myocardial cell apoptosis.

Ginseng has been used in traditional Chinese medicine as a panacea that provides eternal youth [14]. The traditional beneficial effects of ginseng were replenishment of vital energy, mood elevation and longevity [15]. Modern pharmacological studies have revealed the effects of ginseng against cardiovascular diseases associated with aging. Several ginsenosides and ginseng extracts have demonstrated the effects of delaying brain or vascular aging both *in vitro* and *in vivo*. Treatment with fermented *Panax ginseng* extract improved the antioxidant status during aging, thereby minimizing oxidative stress and the occurrence of age-related disorders associated with free radicals [16]. The long-term supplementation of ginseng partially prevented the age-related increase in oxidant production and oxidative protein damage in rats through elevated antioxidant enzyme activities [17]. Ginsenosides could protect cardiomyocyte exposed to hypoxic/ischemic injury through mitochondrial apoptotic pathway. Although most studies provide evidence supporting the heart health properties of ginseng, many controversies have been generated about the cardioprotection of ginseng in middle-aged patients. Until recently, no studies have examined cardiac functional and structural alterations after the long-term consumption of ginseng in intermediate-aged myocardium.

To examine the effects of ginseng on cardiac aging using an *in vivo* system, rodents were selected as an ideal model organism characterized by many significant advantages as an *in vivo* model animal to address the cardiac aging mechanism. Previous studies showed a significant age-dependent decline in left ventricular function and increased prevalence of mitral regurgitation in rat hearts [18, 19]. An increase in apoptosis is involved in the development and progression of cardiac aging. Blocking it by Ginseng can prevent the loss of contractile muscle cells and attenuate cardiac damage [20]. The results of the present study showed that the decrease in infarction size, through RSE treatment in the hearts of both young and intermediate-aged rats, was accompanied by an increase in left ventricular function and a modest reduction in histological changes. RSE gavage for 90 days increased Akt/eNOS activation and Sirt1 and Sirt3 expression but decreased p-Erk expression in intermediate-aged rats. Consistent with this, we found that RSE attenuated age-dependent myocardial apoptosis as displayed by significant reduction of caspase 3/7 activity and Bax/Bcl-2 ratio. Another line of evidence for the protective role of RSE in intermediate-aged heart was demonstrated by inhibition of myocardial MDA production. The accumulation of MDA has been shown to be correlated with increased apoptosis in cardiac aging [21]. Sirt1 was expressed in the nucleus, while Sirt3 was present in mitochondria. The important roles of Sirt1 and Sirt3 in metabolic responses and cardiac aging-related diseases have been extensively studied [22, 23]. While Sirt activators provide benefits as gatekeepers of cellular longevity, the ability of RSE to potentiate these processes remained unknown. The results obtained in the present indicated that cardiac aging paralleled the suppression of Sirt1 and Sirt3, suggesting that RSE protected the intermediate-aged heart through triggering several longevity genes. Activity of Akt has been demonstrated to play a central role in regulating a variety of cardiac cellular processes ranging from survival to aging. Recent studies have shown that sirtuin isoforms Sirt1, Sirt3 and Sirt6 play an essential role in the regulation of Akt activation [24]. Our previous study showed that RSE protected adult heart against I/R via Akt-activated pathway. Therefore, this manuscript elucidated a possible mechanism for the cardioprotective effect of RSE through Sirt 1 and 3/Akt-mediated pathway in cardiac aging. We found for the first time that Sirt1 and Sirt3 were significant down-regulated in intermediate-aged hearts and 90 days RSE treatment mitigated age-dependent decrease. With I/R alone, cardiomyocyte apoptosis plays an imperative role in the development of cardiac aging and Sirt1 and Sirt3 were increased by RSE treatment contributing to hearts tolerance to I/R stress, which suggested that Sirt1 and Sirt3 played an anti-apoptotic role and were involved in the cardioprotection of RSE in intermediate-aged hearts.

The analysis of heart fibrosis according to age subsets showed augmentation with aging [25]. In the present study, we showed increased fibrotic infiltration in intermediate-aged rats compared with young rats with the sham operation. The histological observation revealed increased collagen cross-linking in the endocardium but not in the epicardium, indicating an increase in cardiac fibrosis in middle-aged hearts. After RSE treatment for 90 days, there was no comparable fibrosis in noninfarct and infarct regions. We initially expected that the infarcts would be smaller in middle-aged hearts after RSE treatment, consistent with the reduced collagen content. A potential explanation for this result is that the activation of collagen matrix degradation was triggered through the initiation of the inflammatory response in the infarcted tissue. A stepwise increase in MMP activity has been reported after myocardium infarction [26]. We did not obtain measurements of LV remodeling after acute ischemia reperfusion, potentially indicating the effect of RSE on the extent of collation degradation following myocardium infarct.

Cardiac aging involves a complex network of molecular signals, included mitochondrial oxidative stress, insulin/insulin-like growth factor/PI3K, adrenergic and renin angiotensin II signaling and nutrient signaling pathways [27]. In heart aging, many changes, such as increased cardiomyocyte death [28], extracellular matrix remodeling [29, 30], hypoxia response, angiogenesis [31] and energetic abnormalities [32], predispose the aging heart to the development of heart failure. The anti-aging activity of ginseng was initially supported by evidence showing increased antioxidant enzyme activity. While there is little controversy regarding the effects of antioxidant intervention in cardiac aging, there is a considerable debate concerning whether ginseng protects the aged heart [33].

In conclusion, the results of the present study provided the first demonstration that the long-term consumption of ginseng extract protects intermediate-aged hearts against I/R injury by reducing myocardial infarct size, improving left ventricular function, inhibiting caspase-3/7 activation, decreasing Bax/Bcl-2 ratio and MDA. Ginseng extract potentiated cardioprotective effects through the activation of Akt/eNOS survival signals and the suppression of apoptotic Erk/caspase 7 signaling. In addition, ginseng extract triggered the activation of Sirt1 and Sirt3, suggesting an anti-aging effect. Therefore, we propose that ginseng extract RSE might be used an effective drug for the prevention of aged-related cardiac function deficits and other ventricular changes induced after acute ischemic reperfusion.
